# Reconstruction of Brachial Artery with Great Saphenous Vein Graft in A Case of Residual Soft Tissue Sarcoma Arm

**DOI:** 10.29252/wjps.9.1.88

**Published:** 2020-01

**Authors:** Sumanjit S Boro, Bibhuti Bhusan Borthakur, Vinay Mamidala

**Affiliations:** 1 Department of Plastic Surgery, Dr B. Barooah Cancer Institute, Guwahati, Assam, Branch of Tata Memorial Hospital, Mumbai, India;; 2 Department of Surgical Oncology, Dr B. Barooah Cancer Institute, Guwahati, Assam, Branch of Tata Memorial Hospital, Mumbai, India

**Keywords:** Brachial artery, Resection, Soft tissue, Sarcoma, Great saphenous vein, Graft

## Abstract

Soft tissue sarcomas of the upper extremities are very rare tumors. Due to the complex anatomy of the arm, the management of the soft tissue sarcoma becomes very challenging for the operating surgeons. Nonetheless, a large portion of the patients can be treated in a limb-sparing manner ,if surgical expertises are present .We report a case of 30 years old lady with soft tissue sarcoma of right arm operated in an another hospital, came to our institute with pain in the operated site and positive histological margins. The patient had feeble radial and ulnar artery pulses. We had done a MR angiography of that limb and it showed no flow from mid arm level in the brachial artery, but presence of collaterals around elbow joint. We had removed the residual tumor and also excised 14 cm of right brachial artery. On opening the brachial artery, tumor thrombus was seen along the whole length of the excised segment. The defect was reconstructed with reverse great saphenous vein graft taken from left leg. Post-operative period was uneventful. Doppler ultrasonography done at 6 and 12 months later showed good flow in the grafted segment with minimal narrowing of the anastomosis sites.

## INTRODUCTION

Soft tissue sarcomas are rare malignant mesenchymal tumors with a yearly incidence of less than 1% of newly diagnosed solid tumors.^1^ Overall annual incidence of soft tissue sarcoma is about 2-3 cases per 100,000.^2^ Adult extremity sarcomas are approximately 60% of all soft tissue sarcomas,^3^ and lower limb is more frequently involved than the upper limbs.^4^ Upper extremity involvement is 20% of all soft tissue sarcomas.^5^

Soft tissue sarcoma involving of upper extremity presents a major challenge as the anatomy is complex and functional demand is high and resection margin must be tumor free. Due to high incidence of local recurrence, amputation is the ultimate outcome of most of the upper extremity sarcomas in earlier days.^6^^-^^9^ Nowadays, due to multimodality treatments, limb-saving surgery can be performed in more than 90% of the patients without compromise in local recurrence rates or survival rates.^7^^,^^8^^,^^10^^,^^11^ However, a good surgical resection with adequate tumor clearance remains the mainstay of outcome of soft tissue sarcoma.^6^^,^^7^^,^^12^

## CASE REPORT

This 30 years old lady from a remote village of Assam, India had a mild pain at the right forearm for last 2 years. She developed a small swelling in that region which gradually increased in size. She took treatment from local doctors, but not getting relieved of her pain and swelling. She then came to Guwahati city for better treatment and visited one of the private hospitals. Biopsy was done there along with CT scan. Biopsy came out to be a soft tissue sarcoma. She was operated in that hospital and discharged 5 days after surgery. Immediate post-operative period was uneventful. She again developed pain at the surgical site one month after the surgery. 

She was prescribed pain killers, but not getting relieved of her pain. After 6 months of this on and off pains, she was fade up and came to our institute. She was worked up extensively at our institute by the surgical oncology team. MRI of the arm showed residual disease and haziness around the neuromuscular bundle at the right arm. All pre-anesthetic work ups were done and the patient was put for surgery. Consent was taken regarding the consequences following brachial artery resection including amputation and requirement of great saphenous vein graft.

**Fig. 1:**
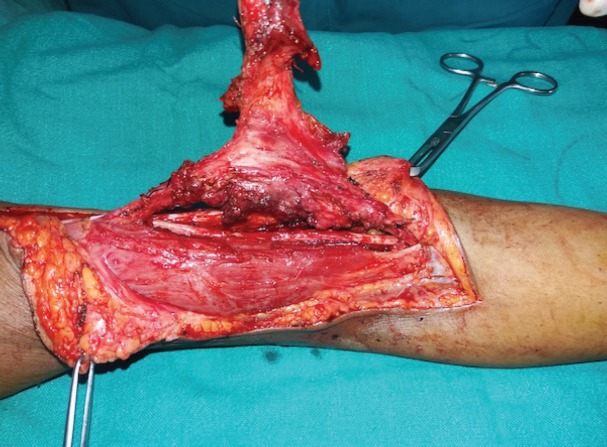
The extent of the soft tissue sarcoma in arm

The patient was intubated and operated under general anesthesia in supine position. Scar of previous surgery was excised. On exploration, it was found that tumor was in close proximity to the neurovascular bundle. On tedious dissection, we were able to separate the tumor from the median nerve, but it was not possible to separate the tumor from the brachial artery. We had to excise 14 cm of the brachial artery. There was tumor thrombus inside the lumen of the brachial artery. After that, we had marked the course of great saphenous vein on the left leg. With careful dissection, we had isolated approximately 16 cm of the great saphenous vein. That segment was cut and placed on a saline filled tray after marking the proximal and distal end (Figure 1).

**Fig. 2:**
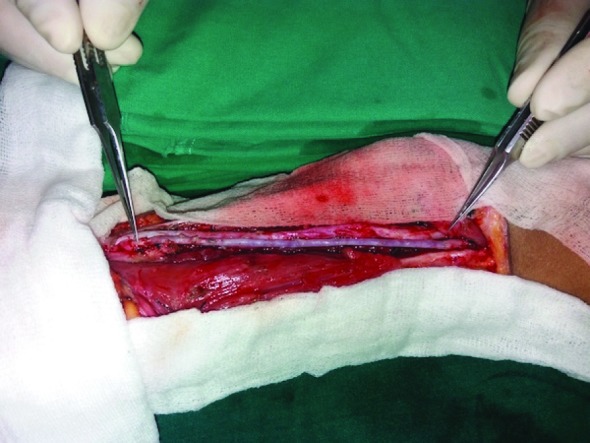
The great saphenous vein graft (in-between the jeweler forceps).

**Fig. 3:**
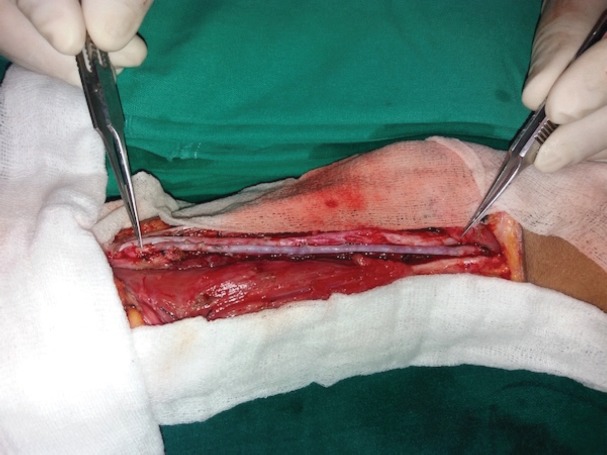
Suture line post-residual tumor excision and brachial artery reconstruction

That isolated vein segment was transferred to the right arm after reversing its ends. First, we had done anastomosis of the proximal end near the shoulder joint and next at distal end near the elbow. Anastomosis was done with 9-0 ethilon round body under microscope (Figure 2). Both ulnar and radial artery pulsations were good at the end of the surgery. The glove drain was put around the anastomosis. Posterior arm slab was applied to restrict any movement of elbow and shoulder joint (Figure 3). In post-operative period, we kept the slab for two weeks and after that arm pouch was advised. Limb elevation was continued for one month. She received chemoradiation following surgery.

We had discharged the patient on 7^th^ post-operative day. For follow up, we advised the patient to come to surgical oncology/plastic surgery OPD at 2^nd^ and 4^th^ week and subsequently at 3^rd^, 6^th^ and 12^th^ month. We carried out a Doppler USG of the right upper limb at 6^th^ and 12^th^ months, which showed good flow at the great saphenous graft with minimal narrowing of anastomosis sites. Patient had no active complaint at the end of 12 months follow up.

## DISCUSSION

Soft tissue sarcomas of extremities are very difficult to manage due to its complex anatomy of arm and the tumor involves many vital structures. Radaelli *et al.* described a series of 105 cases of extremity and retroperitoneal sarcoma enblock excision along with important vessels and reconstruction with vein graft in a span of thirteen years.^13^ They have concluded that vascular resection to facilitate excision of soft tissue sarcoma had an acceptable long term patency rate. Although the encasement of the vascular bundle did not represent a contraindication to surgery, there was an association with a high metastatic risk by virtue of the locally advanced nature of the disease and this should be considered when planning treatment.^13^ Our patient did not have any recurrence during this one year follow up.

Nemoto *et al.* described a series of 15 cases of primary vein graft in free tissue transfers, while the mean length of the grafted vein was 10.8 cm. They mostly used cephalic vein as graft.^14^ Free flap success rate was 100% (15 out of 15) in their study. At follow up, Doppler ultrasonography at 6^th^ and 12^th^ month showed patent graft in our case. Donor site morbidity was significant in cardiac bypass patients. A review of the data in the cardiac registry at The Lehigh Valley Hospital (Allentown, Pennsylvania) showed 0.6% donor site (leg) related complications in hospitalized patients.^15^

It may be due to the poor general condition, prolonged hospital stay and associated comorbidity of these patients. The leg problems have included cellulitis, hematomas or seromas, chronic edema, saphenous neuralgia, hypertrophic and unstable scar.^15^ In our patient, she did not have any such problems during this one year follow up. Vein grafting is an important tool in reconstructive microsurgery. With judicious patient selection, proper technique of graft harvesting and anastomosis, good patient outcome can be achieved.
